# Editorial: Reproduction in aquatic animals

**DOI:** 10.3389/fgene.2023.1127764

**Published:** 2023-01-23

**Authors:** Shubo Jin, Lingbo Ma, Hongtuo Fu

**Affiliations:** ^1^ Wuxi Fisheries College, Nanjing Agricultural University, Wuxi, China; ^2^ Key Laboratory of Freshwater Fisheries and Germplasm Resources Utilization, Ministry of Agriculture and Rural Affairs, Freshwater Fisheries Research Center, Chinese Academy of Fishery Sciences, Wuxi, China; ^3^ Key Laboratory of Marine and Estuarine Fisheries, Ministry of Agriculture and Rural Affairs, East China Sea Fisheries Research Institute, Chinese Academy of Fishery Sciences, Shanghai, China

**Keywords:** aquatic animals, steroid hormone, triploid, reproduction-related genes, reproduction

Reproduction exhibits great diversity in aquatic animals. The maturation of gonads takes a long time in some aquatic animals (ie., Amur sturgeon, *Acipenser schrenckii*) (Qu et al., 2010), while some aquatic animals take only 1–2 months to reach gonad maturation (ie., Oriental river prawn, *Macrobrachium nipponense*) (Jin et al., 2016). Slow gonad development can prolong the reproductive cycle, while rapid gonad development leads to mixing multigenerational populations in a pond. There is also great variation among aquatic animals in egg-laying ability and hatchability. Reproduction is a complicated process consisting of gonad maturation, molting (crustaceans), mating, embryonic development, larval rearing, and so on. At the same time, reproduction is vulnerable to external factors such as diet (Hu et al., 2009), temperature and light (Om et al., 2015; Wedekind, 2017). Therefore, comprehensive research on the genetic basis and underlying mechanisms of aquatic animal reproduction are essential for successful aquaculture seedling production. In recent decades, significant progress has been made in understanding aquatic animal reproduction. Genes, regulatory mechanisms, and externalities associated with reproduction continue to be deciphered. A better understanding of reproduction is very valuable for the development of techniques to control the reproductive process of aquatic animals. The article contents of this Research Topic are shown in Figure 1.

**FIGURE 1:**
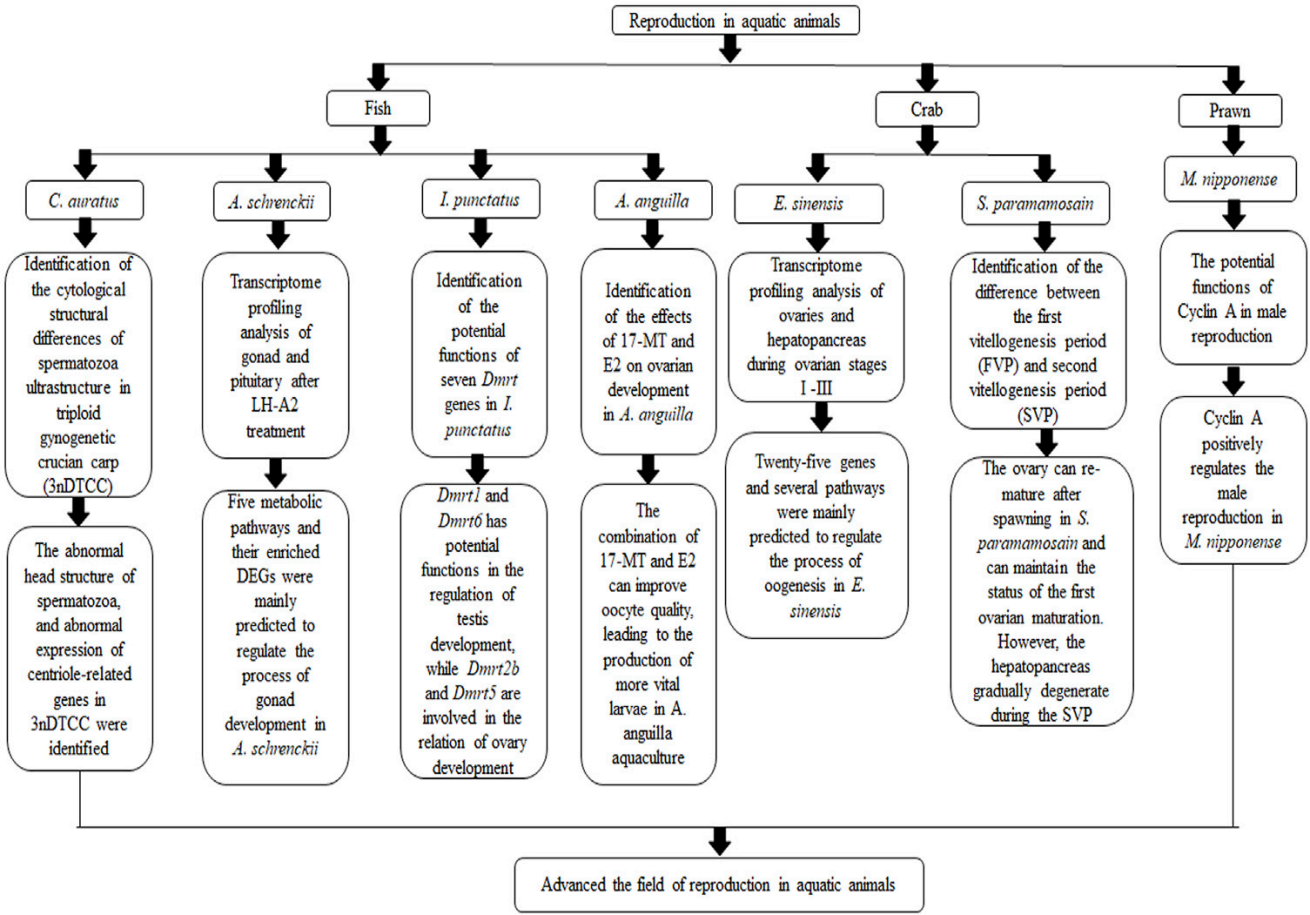
The main article contents of this Research Topic

## 1 Identification of the effects of steroid hormone on gonad development

Steroid hormones can regulate the process of sex determination, gonadal development, and growth in aquatic species *via* interactions with endocrine factors (Couch et al., 1987). The effects of luteinizing hormone releasing hormone A2 (LH-A2) on gonad development in juvenile *A. schrenckii* were determined through performing the transcriptome profiling analysis, treated at a dose of 3 μg/kg. The treatment of LH-A2 resulted in the gradual increase of 17β-estradiol levels, while inhibited the secretion of testosterone. Transcriptome profiling analysis revealed that signal transduction, global and overview maps, immune system, endocrine system and lipid metabolism were the main enriched metabolic pathways of differentially expressed genes (DEGs) in both the pituitary and gonads, predicting these metabolic pathways and the DEGs enriched in these metabolic pathways play essential roles in the regulation of gonad development in *A. schrenckii* (Lv et al.).

This article analysed the possibilities of 17α-methyltestosterone (17-MT) and 17β-estradiol (E2) to replace the pituitary extract (PE) in European silver eels (*AnguillaAnguilla* L.) industry. 17-MT was used as potent androgen to activate the androgen receptor, while E2 can be considered as an inducer of vitellogenesis to shorten the duration of PE treatment. This article identified that 17-MT + E2 group has more significantly regulatory effects on eye index and gonadosomatic index than the other treated groups (17-MT group, E2 group, control group), and the expression of follicle-stimulating hormone receptor in 17 MT + E2 group was 44-folder higher than that of control group, indicating the combination can improve oocyte quality, leading to the production of more vital larvae in *A. anguilla* aquaculture (Palstra et al.).

## 2 Identification of the abnormalities in the spermatozoa of 3nDTCC

The cytological structural differences of spermatozoa ultrastructure between diploid red crucian carp and triploid gynogenetic crucian carp (*Carassius auratus*) (3nDTCC) were analysed. Furthermore, the molecular expression characteristics of the spermatozoa packaging process in 3nDTCC were also determined through measuring the mRNA expression of centriole-related genes, including cep57, *cetn1*, *rootletin*, and *nek2*. This article identified the abnormal head structure of spermatozoa, and abnormal expression of centriole-related genes in 3nDTCC, affecting the motility of spermatozoa, and the normally genetic composition of the gynogenesis offspring (He et al.).

## 3 Identification of reproduction-related genes

Identification of reproduction-related genes plays essential roles in the establishment of artificial technique to regulate the reproductive process in aquatic animals. Seven *Dmrt* (Doublesex and Mab-3 related transcription factor) genes were identified from the genome of the channel catfish (*Ictalurus punctatus*). These *Dmrt* genes included *Dmrt1*, *Dmrt2a*, *Dmrt2b*, *Dmrt3*, *Dmrt4*, *Dmrt5* and *Dmrt6*, and distributed unevenly across five chromosomes. qPCR analysis in different tissues and treatment with 17β-estradiol revealed that *Dmrt1* and *Dmrt6* has potential functions in the regulation of testis differentiation/development, while *Dmrt2b* and *Dmrt5* are involved in the relation of ovary development in this species (Xu et al.).

In invertebrates, the ovaries and hepatopancreas were sampling from the Chinese mitten crab (*Eriocheir sinensis*) during ovarian stages I -Ⅲ, and performed the transcriptome profiling analysis, in order to select the candidate genes and metabolic pathways, involved in the ovarian development. Twenty-five genes and several pathways were mainly predicted to regulate the process of oogenesis in *E. sinensis*. The candidate pathways included the ubiquitin-proteasome pathway, cyclic AMP-protein kinase A signalling pathway, and mitogen-activated protein kinase signalling pathway, playing essential roles in the regulation of ovarian development in *E. sinensis* (Feng et al.).

The differences between the first vitellogenesis period (FVP) and second vitellogenesis period (SVP) in the ovary, hepatopancreas and muscle of mud crab (*Scylla paramamosain*) were determined. This article indicated that the ovary can re-mature after spawning in *S. paramamosain* and can maintain the status of the first ovarian maturation. However, the hepatopancreas gradually degenerate during the SVP (Ren et al.).

The potential functions of *Cyclin A* (*CycA*) in male reproduction were investigated in *M. nipponense*. This article revealed that the ablations of the eyestalk of male *M. nipponense* significantly stimulated the mRNA expressions of *CycA*, and the *CycA* expressions in the testis and androgenic gland taken from the reproductive season were significantly higher than those during the non-reproductive season. Furthermore, knockdown the expressions of *CycA* in male *M. nipponense* by RNAi resulted in the decrease of insulin-like androgenic gland hormone expressions, and the delay of testis development, indicating *CycA* was involved in the regulation of male reproduction in *M. nipponense* (Zhang et al.).
